# Death anxiety among the oldest old in Germany. Evidence from the nationally representative ‘Old Age in Germany (D80+)’

**DOI:** 10.1111/psyg.13200

**Published:** 2024-10-06

**Authors:** André Hajek, Louis Jacob, Supa Pengpid, Karl Peltzer, Razak M. Gyasi, Pinar Soysal, Nicola Veronese, Karel Kostev, Ghazal Aarabi, Hans‐Helmut König

**Affiliations:** ^1^ Department of Health Economics and Health Services Research University Medical Center Hamburg‐Eppendorf, Hamburg Center for Health Economics Hamburg Germany; ^2^ AP‐HP, Université Paris Cité, Lariboisière‐Fernand Widal Hospital Department of Physical Medicine and Rehabilitation Paris France; ^3^ Université Paris Cité, INSERM U1153 Epidemiology of Ageing and Neurodegenerative Diseases (EpiAgeing) Paris France; ^4^ Research and Development Unit Parc Sanitari Sant Joan de Déu, CIBERSAM, ISCIII Barcelona Spain; ^5^ Department of Health Education and Behavioural Sciences, Faculty of Public Health Mahidol University Bangkok Thailand; ^6^ Department of Public Health Sefako Makgatho Health Sciences University Pretoria South Africa; ^7^ Department of Healthcare Administration, College of Medical and Health Science Asia University Taichung Taiwan; ^8^ Department of Psychology University of the Free State Bloemfontein South Africa; ^9^ Department of Psychology, College of Medical and Health Science Asia University Taichung Taiwan; ^10^ African Population and Health Research Centre Nairobi Kenya; ^11^ National Centre for Naturopathic Medicine, Faculty of Health Southern Cross University Lismore New South Wales Australia; ^12^ Department of Geriatric Medicine, Faculty of Medicine Bezmialem Vakif University Istanbul Turkey; ^13^ Geriatrics Section, Department of Internal Medicine University of Palermo Palermo Italy; ^14^ University Hospital Marburg, Philipps‐University Marburg Marburg Germany; ^15^ Department of Periodontics, Preventive and Restorative Dentistry, Centre for Dental and Oral Medicine University Medical Centre Hamburg‐Eppendorf Hamburg Germany

**Keywords:** aged, 80 and above, death anxiety, depression, fear of death, loneliness, oldest old

## Abstract

**Background:**

There is a lack of studies investigating death anxiety among the oldest old based on a large, nationally representative sample during the pandemic. Thus, our aim was to investigate the prevalence and determinants of death anxiety among the oldest old in Germany during the Covid‐19 pandemic.

**Methods:**

Cross‐sectional data were taken from the ‘Old Age in Germany’ (D80+) study. This is a large, nationwide representative study including individuals 80 years and over living at home and individuals in institutionalised settings (*N* = 9542 individuals in the analytic sample).

**Results:**

Overall, 30% of the respondents reported the absence of death anxiety, 45.5% reported a rather not strong death anxiety, 20.2% reported a rather strong death anxiety, and 4.3% reported a very strong death anxiety. Linear regressions revealed that higher death anxiety was significantly associated with being female (β = 0.21, *P* < 0.01), younger age (β = −0.02, *P* < 0.001), being married (β = 0.09, *P* < 0.001), high education (compared to low education, β = 0.07, *P* < 0.05), the presence of meaning in life (β = 0.13, *P* < 0.001), higher loneliness levels (β = 0.18, *P* < 0.001), the presence of multimorbidity (β = 0.07, *P* < 0.05), and poorer self‐rated health (β = −0.07, *P* < 0.001). A further analysis showed that probable depression (β = 0.31, *P* < 0.001) is also associated with higher death anxiety.

**Conclusion:**

About one in four individuals had a strong or very strong fear of death during the pandemic. Several sociodemographic, psychosocial, and health‐related factors are associated with higher death anxiety. This better understanding of the determinants of death anxiety can be relevant for, among others, the affected individuals, informal and professional carers, as well as friends and relatives.

## INTRODUCTION

Individuals aged 80 years and over – often referred to as the ‘oldest old’ – are frequently exposed to the topic of death. For example, some of their friends and relatives may already have died or are dying. Their own health also usually declines significantly during this period of life. This can lead individuals to come to terms with their own mortality. Particularly during the Covid‐19 pandemic (a threat to human existence), when individuals aged 80 years and over had a higher mortality rate from the Covid‐19 infection than younger people, individuals became particularly aware of their own mortality.^1^ This can lead to death anxiety (also known as ‘fear of death’), referring to an ‘emotional reaction provoked by anticipation of death generated by perceptions of a real or imaginary threat to the existence of one's own’^2^ (p. 225).

Death anxiety is associated with low well‐being,^3^ poor mental health,^4^ and suicidality.^5^ On the other hand, fear of death can also lead to living life more consciously and savouring life to the full, leading to positive outcomes. However, we assume that fear of death was more likely to be associated with negative outcomes during the pandemic, particularly due to pandemic‐related restrictions (e.g., travelling, contact restrictions, curfews).^1^

Due to its potential consequences for well‐being and suicidality, knowledge about the prevalence and determinants of death anxiety is of great importance. Thus far, former research has focused on death anxiety in specific groups, such as individuals with cancer^6^ or individuals with HIV/AIDS.^7^ For example, a recent meta‐analysis showed that higher death anxiety was, among other things, associated with being female, worse mental health, younger age, low education, or low sense of meaning in life among individuals with cancer.^6^ It should be noted that such individual factors may overlap or interact.^8^ Additionally, they encompass aspects such as a person's spirituality, former life experiences, cultural standards, social support, and the surrounding environment.^8^

Patra *et al*.^9^ conducted a systematic review and meta‐analysis on death anxiety during the times of the Covid‐19 pandemic. They found a death anxiety score of 42.9% among the general population, whereas the score was 56.4% (95% CI: 52.7% to 60.1%) among older adults. The existing four studies^10^, ^11^, ^12^, ^13^ included in this meta‐analysis^9^ dealing with death anxiety among older adults exclusively used data from Turkey and the United Arab Emirates. Moreover, the four studies are united by the fact that they are based on selective samples between 100 and 350 older individuals.^10^, ^11^, ^12^, ^13^ In her recent meta‐analysis, Letzner^4^ also pointed out that ‘expanding and diversifying the age range up to 80+ years old would be noteworthy’ (p. 404). Due to the lack of studies investigating death anxiety among the oldest old based on a large, nationally representative sample during the pandemic, our aim was to investigate the prevalence and determinants of death anxiety among the oldest old in Germany in this period of time. Using nationally representative data also ensures the generalisability of our findings.

Knowledge about the determinants of death anxiety during the Covid‐19 pandemic is of great importance – particularly in the light of potential future pandemics. It may also be of importance for private and professional caregivers to provide better emotional support. This may improve the satisfaction of individuals susceptible to high death anxiety. Furthermore, some factors associated with death anxiety are modifiable (e.g., loneliness) and have the potential to alter death anxiety. This, in turn, is important because – as noted earlier – death anxiety can contribute to suicidal ideation^5^ and low well‐being.^3^

## METHODS

### Sample

Data were taken from the ‘Old Age in Germany’ (D80+) study, which reflected a large, nationally representative sample of individuals aged 80 years and over living in Germany. Individuals residing in the community as well as those living in institutionalised settings, such as nursing homes, were included in the D80+ study.

The University of Cologne conducted the D80+ study in cooperation with the Cologne Centre for Ethics, Rights, Economics, and Social Sciences of Health (ceres) and the German Centre of Gerontology (DZA). Funding was provided by the Federal Ministry for Family Affairs, Senior Citizens, Women and Youth (BMFSFJ). A well‐known institute for market and social research (Infas) conducted the data collection.

Due to the pandemic, written questionnaires (rather than initially planned face‐to‐face interviews) were used. Data were collected from November 2020 to April 2021. Albrecht *et al*.^14^ provided more details about the D80+ study. The analytic sample equalled *N* = 9542 individuals.

The ethics board of the Medical Faculty at the University of Cologne approved the D80+ study (Protocol #: 19‐1387_1). The D80+ study is in line with the Helsinki Declaration. A short introduction and the privacy statement were included in the questionnaire. When respondents completed and returned the questionnaire, consent was provided.

### Outcome

In line with prior research,^15^ death anxiety was assessed using a common single‐item measure. The exact wording was: ‘How strong is your fear of your own dying?’ Four response categories were given: 1 = not strong at all; 2 = rather not strong; 3 = rather strong; 4 = very strong). Such single‐item assessments are quite reliable across time and correlate moderately with multi‐item tools.^16^

### Independent variables

Following past research,^10^, ^11^, ^12^, ^13^ determinants were selected for regression analysis. More precisely, sociodemographic factors were included as follows in regression analysis: Sex (men; women), age, family status (five categories: married, living together with spouse; married, living separated from spouse; divorced; widowed; single), presence of children (no; yes), living arrangement (private household; institutionalised setting), and education (following the ISCED‐11 classification:^17^ low education; medium education; high education). The presence of physical activity (no; yes) was added as a lifestyle factor in regression analysis. Meaning in life (no; neither; yes; dichotomised into: no/neither or yes) and loneliness (single item ranging from 1 = never or almost never to 4 = always or almost always, whereby higher scores reflect higher loneliness levels) were added as psychosocial factors in regression analysis.

Health‐related factors were included as follows in regression analysis. Self‐rated health is based on a single‐item measure with four response options (from 1 = very bad to 4 = very good), functional impairment is based on a modified version of the Instrumental Activities of Daily Living (IADL) created by Lawton *et al*.^18^ It consists of seven items. By averaging the items, the final score was created. This final score ranges from 0 to 2, with higher values reflecting higher functional impairment. As is common, multimorbidity was defined as the presence of two or more chronic conditions. To this end, 21 chronic conditions, such as stroke or cancer, were considered. The chronic conditions are based on the multimorbidity index in old age.^19^, ^20^

In further analysis, probable depression was quantified using the ‘Short Form of the Depression in Old Age Scale’ (DIA‐S4), which has favourable psychometric properties.^21^, ^22^ The score of the DIA‐S4 ranges from 0 to 4, whereby higher values reflect more depressive symptoms. A cut‐off score of 1.5 was used to indicate probable depression following former research.^23^ Probable depression was only included in this further model due to the unclear directionality^4^ between probable depression and death anxiety. In an additional model, it was also assessed for the factor of how the Covid‐19 pandemic has influenced one's health (from 1 = not at all to 5 = very strong).

### Statistical analysis

First, the characteristics of the analytic sample are shown. Thereafter, the prevalence of death anxiety among the total sample and for certain subgroups (for example, by age groups, sex, marital status, living arrangement, or education) is provided. Subsequently, multiple linear regressions were used to investigate the determinants of death anxiety. Cluster‐robust standard errors (clustered at the individual level) were calculated. Sampling weights were used to account for the sampling design and non‐response.^24^

In sensitivity analyses, ordered logistic regressions were used rather than linear regressions. Listwise deletion was first used. However, to account for missing data, a full‐information maximum likelihood (FIML) approach^25^ was additionally used. Variance inflation factors (VIFs) were calculated. However, the average VIF was 1.42 (with the highest VIF equalling 2.10), indicating the absence of multicollinearity. Statistical significance was set at *P* < 0.05 in this study. Statistical analyses were performed using Stata 18.0 (Stata Corp., College Station, TX, USA).

## RESULTS

### Sample characteristics and prevalence

Sample characteristics for the weighted analytic sample (n = 9542 individuals) are shown in Table 1. The average age equalled 85.5 years (SD: 4.1 years, 80 to 100 years), with 61.8% of the individuals being female. Moreover, 90.0% of the individuals resided in a private household. More details are displayed in Table 1.

**Table 1:** Sample characteristics (analytic sample, with *N* = 9542 individuals, weighted)

Variables	*n* (%)/mean (SD)
Sex	
Men	3650 (38.2%)
Women	5892 (61.8%)
Age, years	85.5 (4.1)
Marital status	
Married, living together with spouse	3831 (40.5%)
Other: married, living separated from spouse; widowed; divorced; single	5622 (59.5%)
Presence of children	
No	954 (10.0%)
Yes	8588 (90.0%)
Education	
Low education	2106 (22.8%)
Medium education	4787 (51.8%)
High education	2341 (25.4%)
Living situation	
Private household	8584 (90.0%)
Institutionalised setting	958 (10.0%)
Physical activity	
No	4134 (43.3%)
Yes	5408 (56.7%)
Meaning in life	
Neither/no	1979 (21.5%)
Yes	7237 (78.5%)
Loneliness, 1 to 4, higher values reflect higher loneliness levels	1.7 (0.8)
Multimorbidity, two or more chronic conditions	
Absence of multimorbidity	960 (10.2%)
Presence of multimorbidity	8440 (89.8%)
Self‐rated health, 1 to 4, higher values reflect a more favourable self‐rated health	2.6 (0.7)
Functional impairment, 0 to 2, higher values reflect a higher functional impairment	0.6 (0.7)
Probable depression	
Absence of probable depression	5180 (59.6%)
Presence of probable depression	3506 (40.4%)
Effect of the pandemic on one's own health, 1 to 5, higher values reflect stronger influence	1.4 (0.8)

In Table 2, prevalence rates are shown (total sample), as well as by certain important subgroups. Overall, 30% of the respondents reported the absence of death anxiety, 45.5% reported a rather not strong death anxiety, 20.2% reported a rather strong death anxiety, and 4.3% reported a very strong death anxiety. Particular high differences in the prevalence rate were present between individuals with probable depression (e.g., strong or very strong death anxiety: 34.8%) and individuals without probable depression (strong or very strong death anxiety: 17.3%). Additional details are depicted in Table 2.

**Table 2:** Prevalence rates (total sample and for certain groups; weighted)

	Death anxiety (95% CI)			
Group	Not strong at all	Rather not strong	Rather strong	Very strong
Total sample	30.0% (29.0%–31.1%)	45.5% (44.4%–46.7%)	20.2% (19.2%–21.1%)	4.3% (3.8%–4.8%)
Sex				
Male	31.8% (30.3%–33.4%)	46.8 (45.1%–48.4%)	18.2% (17.0%–19.5%)	3.2% (2.7%–3.9%)
Female	28.9% (27.5%–30.4%)	44.8 (43.2%–46.4%)	21.4% (20.1%–22.7%)	4.9% (4.3%–5.7%)
Age group				
80–84	29.0% (27.6%–30.4%)	46.0% (44.5%–47.5%)	20.6% (19.3%–21.8%)	4.5% (3.9%–5.2%)
85–89	31.3% (29.4%–33.3%)	44.3% (42.3%–46.4%)	20.2% (18.7%–21.9%)	4.2% (3.4%–5.1%)
90+	32.1% (29.6%–34.8%)	45.8% (42.9%–48.7%)	18.4% (16.3%–20.7%)	3.7% (2.8%–5.0%)
Family status				
Married	28.1% (26.7%–29.7%)	47.4% (45.8%–49.1%)	20.8% (19.4%–22.2%)	3.7% (3.1%–4.3%)
Other^†^	31.1% (29.6%–32.6%)	44.3% (42.6%–45.9%)	20.0% (18.7%–21.3%)	4.7% (4.1%–5.4%)
Living arrangement				
Private household	29.8% (28.8%–30.8%)	46.0% (44.9%–47.2%)	19.9% (19.0%–20.9%)	4.3% (3.8%–4.8%)
Institutionalised setting	32.2% (27.9%–36.7%)	41.1% (36.4%–45.9%)	22.3% (18.4%–26.7%)	4.5% (2.7%–7.4%)
Education				
Low education	31.7% (29.4%–34.1%)	44.7% (42.0%–47.4%)	17.9% (16.1%–19.9%)	5.7% (4.7%–7.0%)
Medium education	30.1% (28.6%–31.6%)	44.4% (42.7%–46.0%)	21.2% (19.9%–22.5%)	4.4% (3.7%–5.2%)
High education	28.2% (26.3%–30.2%)	48.6% (46.5%–50.8%)	20.4% (18.7%–22.3%)	2.7% (2.1%–3.5%)
Region				
West Germany	30.0% (28.8%–31.3%)	46.3% (45.0%–47.7%)	19.8% (18.8%–20.8%)	3.9% (3.4%–4.4%)
East Germany	30.0% (28.2%–31.9%)	42.7% (40.5%–44.9%)	21.6% (19.5%–23.8%)	5.8% (4.6%–7.3%)
Citizenship				
Other citizenship	30.5% (22.7%–39.5%)	48.8% (39.6%–58.2%)	16.1% (10.1%–24.9%)	4.6% (1.9%–10.5%)
German citizenship	30.2% (29.1%–31.3%)	45.6% (44.4%–46.8%)	20.1% (19.2%–21.0%)	4.2% (3.7%–4.7%)
Depression				
Absence of probable depression	33.8% (32.4%–35.2%)	49.0% (47.5%–50.5%)	15.2% (14.2%–16.3%)	2.1% (1.7%–2.5%)
Presence of probable depression	23.9% (22.2%–25.6%)	41.4% (39.3%–43.5%)	27.0% (25.4%–28.8%)	7.8% (6.7%–9.0%)
Multimorbidity				
Absence of multimorbidity	36.8% (33.5%–40.3%)	44.6% (41.3%–47.9%)	15.1% (12.9%–17.6%)	3.5% (2.5%–4.9%)
Presence of multimorbidity	29.2% (28.1%–30.3%)	45.7% (44.4%–46.9%)	20.8% (19.8%–21.8%)	4.4% (3.9%–4.9%)

^†^ Other includes: married, living separated from spouse, divorced, widowed, single.

### Determinants of death anxiety

In Table 3, the findings of regression analyses are shown (second column: with listwise deletion to handle missing data; third column: with FIML to handle missing data). We particularly refer to the regressions with FIML to address missing data in this section. However, it should be emphasised that both (i.e., models with listwise deletion and models with FIML) produced very similar results (in terms of significance and effect size).

**Table 3:** Determinants of death anxiety: results of linear regressions

Independent variables	Death anxiety (with listwise deletion to handle missing data)	Death anxiety (with full‐information maximum likelihood to handle missing data)
Sex: women (reference category: men)	0.22**	0.21**
	(0.08–0.36)	(0.08–0.35)
Age	−0.02***	−0.02***
	(−0.03 to −0.01)	(−0.03 to −0.01)
Marital status: married (reference category: other^†^)	0.09***	0.09***
	(0.04–0.14)	(0.05–0.13)
Presence of at least one child: yes (reference category: no)	0.02	0.03
	(−0.05–0.09)	(−0.04–0.10)
Living arrangement: institutionalised setting (reference category: private household)	−0.09+	−0.06
	(−0.18–0.01)	(−0.16–0.03)
Education		
Medium (reference: low)	0.06*	0.04
	(0.00–.11)	(−0.01–0.09)
High	0.09**	0.07*
	(0.02–0.15)	(0.01–0.13)
Sports activity: yes (reference category: no)	−0.02	−0.02
	(−0.06–0.03)	(−0.07–0.02)
Meaning in life: yes (reference category: neither/no)	0.14***	0.13***
	(0.08–0.19)	(0.08–0.18)
Loneliness (1 = never/almost never to 4 = almost or almost always, with higher values reflecting higher loneliness levels)	0.18***	0.18***
	(0.15–.21)	(0.14–0.21)
Multimorbidity (i.e., at least two chronic conditions): yes (reference category: no)	0.09**	0.07*
	(0.03–0.15)	(0.01–0.13)
Self‐rated health (1 = very bad to 4 = very good)	−0.06**	−0.07***
	(−0.10 to −0.02)	(−0.10 to −0.03)
Functional impairment (Instrumental Activities of Daily Living; ranging from 0 to 2, with higher values reflect higher functional impairment)	0.04+	0.03
	(−0.01–0.08)	(−0.01–0.07)
Observations	8682	9542
*R* ^2^	0.04	0.04

*** *P* < 0.001;

** *P* < 0.01;

* *P* < 0.05;

^+^ *P* < 0.10.

^†^ Other includes: married, living separated from spouse, divorced, widowed, single.

Unstandardised beta coefficients are displayed; 95% CI in parentheses; cluster‐robust standard errors were calculated; weights were used; adjusted for sample cells (used for the stratification of the secondary sampling unit).

Linear regressions revealed that higher death anxiety was significantly associated with being female (β = 0.21, *P* < 0.01), younger age (β = −0.02, *P* < 0.001), being married (β = 0.09, *P* < 0.001), high education (compared to low education, β = 0.07, *P* < 0.05), the presence of meaning in life (β = 0.13, *P* < 0.001), higher loneliness levels (β = 0.18, *P* < 0.001), the presence of multimorbidity (β = 0.07, *P* < 0.05), and poorer self‐rated health (β = −0.07, *P* < 0.001). In another model, linear regressions were replaced by ordinal logistic regressions (see Table S1). Findings remained virtually the same in terms of significance.

In a further analysis, probable depression was added as an independent variable (see Table 4). The findings were mostly comparable. It should be noted that the beta coefficient for loneliness was somewhat attenuated but remained significant. Probable depression was associated with higher death anxiety (β = 0.31, *P* < 0.001), whereas the significant association between poorer self‐rated health and higher death anxiety disappeared in this model.

**Table 4:** Determinants of death anxiety: results of linear regressions (with probable depression as independent variable)

Independent variables	Death anxiety (with listwise deletion to handle missing data)	Death anxiety (with full‐information maximum likelihood to handle missing data)
Sex: women (reference category: men)	0.18*	0.19**
	(0.04–0.32)	(0.06–0.33)
Age	−0.02**	−0.02**
	(−0.03 to −0.01)	(−0.03 to −0.01)
Marital status: married (reference category: other^†^)	0.08**	0.08***
	(0.03–0.13)	(0.03–0.12)
Presence of at least one child: yes (reference category: no)	0.02	0.02
	(−0.06–0.09)	(−0.04–0.09)
Living arrangement: institutionalised setting (reference category: private household)	−0.08	−0.06
	(−0.18–0.02)	(−0.15–0.03)
Education		
Medium (reference: low)	0.04	0.04+
	(−0.01–0.10)	(−0.01–0.09)
High	0.08*	0.07*
	(0.01–0.14)	(0.01–0.14)
Sports activity: yes (reference category: no)	−0.02	−0.03
	(−0.07–0.02)	(−0.07–0.02)
Meaning in life: yes (reference category: neither/no)	0.20***	0.19***
	(0.14–0.25)	(0.14–0.24)
Loneliness (1 = never/almost never to 4 = almost or almost always, with higher values reflecting higher loneliness levels)	0.12***	0.12***
	(0.08–0.16)	(0.09–0.16)
Multimorbidity (i.e., at least two chronic conditions): yes (reference category: no)	0.08**	0.07*
	(0.02–0.15)	(0.01–0.13)
Self‐rated health (1 = very bad to 4 = very good)	−0.01	−0.02
	(−0.05–0.03)	(−0.05–0.02)
Functional impairment (Instrumental Activities of Daily Living; ranging from 0 to 2, with higher values reflect higher functional impairment)	0.01	−0.00
	(−0.03–0.06)	(−0.04–0.04)
Probable depression: yes (reference category: no)	0.32***	0.31***
	(0.27–0.37)	(0.27–0.36)
Observations	8010	9542
*R* ^2^	0.06	0.06

*** *P* < 0.001;

** *P* < 0.01;

* *P* < 0.05;

^+^ *P* < 0.10.

^†^ Other includes: married, living separated from spouse, divorced, widowed, single.

Unstandardised beta coefficients are displayed; 95% CI in parentheses; cluster‐robust standard errors were calculated; weights were used; adjusted for sample cells (used for the stratification of the secondary sampling unit).

In another model, the factor of how the Covid‐19 pandemic has influenced one's health was added to our regression model. A stronger influence of one's own health due to the pandemic was associated with higher death anxiety (β = 0.04, *P* < 0.01). The other findings remained nearly the same in terms of effect size and significance.

## DISCUSSION

The aim of this study was to investigate the prevalence and determinants of death anxiety among the oldest old in Germany during the Covid‐19 pandemic. We have shown that death anxiety is strong or very strong in about one in four individuals. Some sociodemographic, psychosocial, and health‐related factors are associated with higher death anxiety. Based on data from a large, nationally representative sample, our present study markedly extends our current understanding in this research area based on small, selective samples conducted during the Covid‐19 pandemic.

Our reported prevalence rates are lower compared to the death anxiety scores reported by a previous meta‐analysis.^9^ However, this former meta‐analysis converted the average score (raw score) to a standard score. Due to these methodological differences, these rates are hardly comparable. Conducted during the pandemic and using a single‐item tool (five response categories from 1 = disagree strongly to 5 = agree strongly; item: ‘I am afraid of death’), a former study^26^ showed that 26.3% agree ‘a little’ and 18.8% agreed ‘strongly’ to this item (453 adult MTurk workers in the United States). Such prevalence rates are roughly comparable. The high rate of individuals reporting strong death anxiety may be explained by the fact that data collection took place in a very early stage of the Covid‐19 pandemic (April 15, 2020).

With regard to sociodemographic factors, we found that higher death anxiety was significantly associated with being female, having a younger age, being married, and having a high education. This is partly (female and younger age) in accordance with previous research.^27^ Former research has already demonstrated the link between being female and higher death anxiety.^27^ This is often attributed to a higher tendency to worry among women.^28^ The factor that women outlive their husbands and then die without their partners may also be of importance here. Following the death anxiety model suggested by Tomer and Eliason,^29^ regret about the future may be particularly strong among individuals aged 80 years (e.g., compared to individuals 95 years old) because the former group has even more lifetime to lose. Death could also lose some of its horror in very old age, as one has already seen many friends and relatives die. Death could also be seen in part as a release from the discomforts of old age.

The fact that highly educated people (compared to individuals with low education) have a greater fear of death in our study may be explained by the fact that they may have more to lose in their view (such as friends and family still alive, various cognitive activities, travelling).^30^ Such factors may be less pronounced in less educated individuals as many friends may have already died, which may reflect a lower death anxiety among individuals with low education. Moreover, a high death anxiety among married individuals may be explained in particular by the fact that these individuals are afraid of being separated by death from their partner^31^ – who they presumably have been with for a long time.

With regard to psychosocial factors, the presence of meaning in life and higher loneliness were associated with higher death anxiety. This might seem counterintuitive at first glance, as a meaning in life might seem reassuring (with a potential anxiety‐relieving effect)^32^, ^33^. However, from our point of view, such findings are very plausible, as meaning in life could also be linked to fears of loss, for example, the realisation that this meaningful life may one day come to an end. A sense of purpose can also reflect valuable friendships and relationships with relatives that he or she does not want to give up and are afraid of losing. The association between higher loneliness levels and higher death anxiety may particularly reflect the fact that individuals do not want to die alone but rather long for warmth, contact, and security at the end of their lives.^34^

In terms of health‐related factors, poorer self‐rated health and the presence of multimorbidity were associated with higher death anxiety in our study. Generally, poorer health is likely to make individuals even more aware of their own mortality – especially in pandemic times.^1^ This may contribute to a higher death anxiety. Moreover, a former meta‐analysis also showed a strong association between mental health and death anxiety^4^ – aligning with our results. Such an association is highly plausible. For example, depression is associated with a negative outlook on life and is also associated with pain.^35^, ^36^ Both factors may increase the awareness of their own mortality, which in turn can contribute to a higher death anxiety.

When interpreting our present findings, some strengths and shortcomings should be kept in mind. Data were taken from a very large sample representing the population aged 80 and over residing in Germany. Both individuals residing in private households and those living in institutionalised surroundings were included. Death anxiety was assessed using a single‐item measure with a high face validity.^16^ Nevertheless, future research could use multi‐item tools to better capture the complexity of death anxiety. One shortcoming of the D80+, particularly in terms of directionality, is its cross‐sectional design. Other factors (e.g., trust in political parties) could also be of relevance and should be examined in upcoming research.

In conclusion, one in four individuals aged 80 years and over had a strong or very strong fear of death in Germany during the Covid‐19 pandemic. A variety of factors (sociodemographic, psychosocial, and health‐related factors) were associated with higher death anxiety. This may help to provide targeted support for people at risk for high levels of death anxiety. This better understanding of the determinants of death anxiety can be relevant for, among others, the affected individuals, informal and professional carers, as well as friends and relatives. Future research using longitudinal data would be beneficial. Comparisons between countries may also be of interest.

## FUNDING INFORMATION

None.

## CONFLICT OF INTEREST STATEMENT

The authors declare the research was conducted in the absence of any commercial or financial relationships that could be construed as a potential conflict of interest.

## ETHICS STATEMENT

The ethics board of the Medical Faculty at the University of Cologne (Protocol #: 19‐1387_1) approved the D80+ study. The D80+ study is in line with the Helsinki Declaration. The interviews were only conducted with the consent of the interviewees. The questionnaire itself contains a brief introduction and the privacy policy. Consent is given when the respondents complete and return the questionnaire.

## Supporting information


**Supplementary Table S1.** Determinants of death anxiety: results of ordinal logistic regressions.

## Data Availability

All data are available from the German Centre of Gerontology. For further details (application for data use): https://www.dza.de/en/research/fdz/access-to-data/application.
