# Supplementary material for: Death anxiety among the oldest old in Germany. Evidence from the nationally representative ‘Old Age in Germany (D80+)’
Source: Psychogeriatrics. 2024 Oct 6;24(6):1347–55. doi: 10.1111/psyg.13200 (PMC12997142; doi:10.1111/psyg.13200)
Supplement: Supplementary file 1 — Supplementary Table S1. Determinants of death anxiety: results of ordinal logistic regressions. [file PSYG-24-1347-s001.docx]

Supplementary Table 1. Determinants of death anxiety: Results of ordinal logistic regressions

| Independent variables | Death anxiety |
| --- | --- |
|  |  |
| Sex: Women (Reference category: Men) | 1.78*** |
|  | (1.28 - 2.47) |
| Age | 0.95*** |
|  | (0.92 - 0.97) |
| Marital status: Married (Reference category: Other^#^) | 1.25*** |
|  | (1.12 - 1.40) |
| Presence of at least one child: Yes (Reference category: No) | 1.05 |
|  | (0.89 - 1.25) |
| Living arrangement: Institutionalized setting (Reference category: Private household) | 0.82 |
|  | (0.65 - 1.04) |
| Education: - Medium (Reference: Low) | 1.15* |
|  | (1.02 - 1.30) |
| - High | 1.27** |
|  | (1.10 - 1.48) |
| Sports activity: Yes (Reference category: No) | 0.97 |
|  | (0.88 - 1.08) |
| Meaning in life: Yes (Reference category: Neither/No) | 1.40*** |
|  | (1.23 - 1.58) |
| Loneliness (1 = never/almost never to 4 = almost or almost always, with higher values reflecting higher loneliness levels) | 1.52*** |
|  | (1.40 - 1.65) |
| Multimorbidity (i.e., at least two chronic conditions): Yes (Reference category: No) | 1.26** |
|  | (1.09 - 1.46) |
| Self-rated health (1 = very bad to 4 = very good) | 0.88** |
|  | (0.80 - 0.96) |
| Functional impairment (IADL; ranging from 0 to 2, with higher values reflect higher functional impairment) | 1.08 |
|  | (0.97 - 1.19) |
| Pseudo R² | .02 |
| Observations | 8,682 |

*** p<0.001, ** p<0.01, * p<0.05, + p<0.10; odds ratios are displayed; 95% CI in parentheses; cluster-robust standard errors were calculated; weights were used; adjusted for sample cells (used for the stratification of the secondary sampling unit).

# Other includes: Married, living separated from spouse, Divorced, Widowed, Single
